# Sources of health information among U.S. cancer survivors: results from the health information national trends survey (HINTS)

**DOI:** 10.3934/publichealth.2020031

**Published:** 2020-06-12

**Authors:** Inimfon Jackson, Ikponmwosa Osaghae, Nnenna Ananaba, Aniekeme Etuk, Nsikak Jackson, Onyema G Chido-Amajuoyi

**Affiliations:** 1 Department of Epidemiology, Human Genetics and Environmental Sciences, University of Texas School of Public Health, University of Texas Health Science Center at Houston, Houston, TX, USA; 2 Department of Health Promotion and Behavioral Sciences, University of Texas School of Public Health, University of Texas Health Science Center at Houston, Houston, TX, USA; 3 Department of Management, Policy and Community Health, University of Texas School of Public Health, University of Texas Health Science Center at Houston, Houston, TX, USA; 4 Department of Epidemiology, University of Texas MD Anderson Cancer Center, Houston, TX, USA

**Keywords:** cancer, cancer survivorship, health information source, health information-seeking, health communication

## Abstract

**Background:**

Health information is crucial for preservation of health and maintenance of healthy practices among cancer survivors. This study examines the sources and factors associated with choice of health information source among cancer survivors and those without a cancer history.

**Methods:**

We examined health information sources utilized by cancer history between 2011–2014 and 2017–2018 using the Health Information National Trends Survey (HINTS). Factors associated with seeking health information were examined using multinomial logistic regression. Data from HINTS 4, cycles 1–4 (2011–2014) and HINTS 5, cycles 1–2 (2017–2018) were combined and used for all analyses. HINTS-FDA, cycles 1–2 (2015–2017) were excluded from this study because the question about a cancer history was not asked.

**Results:**

Over half of cancer survivors (52.7%) and those without a cancer history (60.9%) obtained their health information through the media. Among cancer survivors, factors associated with health information seeking either through the media or interpersonal communication relative to not seeking information were age, gender, level of education, income, marital status and having a regular healthcare provider. Male survivors were 39% less likely to seek health information from the media (aOR: 0.61; 95% CI: 0.38–0.99) while those with a regular health provider had significantly higher odds of seeking health information via interpersonal communication (aOR: 1.92; 95% CI: 1.09–3.38). In addition, widowed cancer survivors had lower odds of seeking health information from either interpersonal communication (aOR: 0.28; 95% CI: 0.13–0.60) or the media (aOR: 0.30; 95% CI: 0.13–0.69). In the study population without a cancer history, compared to non-Hispanic whites, non-Hispanic blacks, Hispanics and non-Hispanic other categories were significantly less likely to seek health information from the media rather than not seek health information.

**Conclusion:**

Socioeconomic status, marital status, gender and age are important correlates of choice of health information source among cancer survivors in the US. These factors may be useful in guiding interventions aimed at various groups of cancer surviving populations to ensure that they improve their health seeking behaviors.

## 1. Background

The population of cancer survivors in the US is on the rise [1]. In 2019, about 17 million cancer survivors were documented in the US, with this number projected to exceed 22 million by 2030 [2]. The increasing cancer survival rates can be largely attributed to advances in screening, early diagnoses, and treatment of cancers as well as a growing and aging US population [2],[3]. Approximately 45% of cancer survivors are alive 10 years or more after diagnosis, which implies that a significant proportion of this population is exposed to the physical, emotional and psychological trauma that accompanies their condition, together with the long-term adverse effects of cancer therapy such as radiotherapy [1]. This phase is understandably associated with a tendency to seek health information in order to allay the fears and concerns associated with the diagnosis of cancer for both the patient and their caregivers [4].

Most cancer patients tend to seek health information beyond that provided by their physician [5]. Regardless of patients' satisfaction with their physician, they may seek additional health information from other available sources [5]. Nearly 50% of Americans and over 60% of cancer survivors seek cancer-related information from at least one source [6] to allay the anxiety, economic and psychological effect associated with a cancer diagnosis. In addition, the source and volume of health information readily accessible to patients has increased in recent times due to advances in technology and media [6]. Media (including print, radio, tv, and internet) and interpersonal communication, including healthcare providers, are notable sources of health information. Recently, the internet is by far the most common source of adult information among the various media sources in the US [6]. While cancer survivors are more likely to seek health information from their healthcare providers, they overwhelmingly resort to the internet as their first point of call even before their healthcare providers [7]. Media use has especially positive effects on individuals who do not engage much in interpersonal communication [8]. This means that individuals with low levels of interpersonal communication, who are less likely to engage in healthy lifestyle behaviors, may have a higher likelihood of living more healthy lives by information they get from media sources like the internet and television [8].

Previous research has been conducted using nationally representative data and differences in health information seeking behaviors among cancer survivors are well documented. These differences have been shown to be distributed along racial, demographic and socioeconomic lines. Relative to health information seeking from healthcare providers, survivors seeking health information from the internet have been found to be younger and more educated [7]. A different study also found that cancer survivors who used the internet were more likely to be male, younger, and have a higher level of education [9]. Furthermore, factors such as age, gender, level of education and having a regular health provider have been reported to determine survivors' health information seeking. Older patients and females were reported to be more likely to seek health information while the less educated and those with a regular health provider were less likely to do the same [6].

Previous studies have explored the factors associated with seeking health information among cancer survivors by comparing seekers to non-seekers. However, no recent study has specifically considered factors that affect health information seeking via the media and interpersonal communication compared to not seeking health information. Furthermore, associated factors have not been looked at among cancer survivors seeking information from these specific sources to examine how these differ from factors among those without a cancer diagnosis. This study aims to examine the factors associated with different sources of health information among cancer survivors compared to individuals without a cancer history using a nationally representative sample of adults participating in the Health Information National Trends Survey (HINTS). Understanding factors that predict health information seeking through these different sources will improve health communication interventions and focus them towards groups of survivors that will benefit most from them.

## 2. Methods

### 2.1. Data

Participants included in this study were respondents of the Health Information National Trends Survey (HINTS). HINTS is a nationally representative probability cross-sectional study of adults aged 18 or older in the civilian, non-institutionalized population of the United States. It assesses the trends in understanding, usage, and access to health-related information. HINTS collects data about the use of health-related and cancer-related information. Since the same algorithm was used to generate final weights across all the cycles, we appended the cycles and used their associated weights. Our data analyses were based on data from 20,365 respondents from HINTS 4, cycles 1–4 (2011–2014) and HINTS 5, cycles 1–2 (2017–2018). HINTS-FDA, cycles 1–2 (2015–2017) were excluded from this study because the question about a cancer history was not asked. Further information on the survey development, methodology and design have been published previously [3],[6],[7]. HINTS questionnaires, data, and reports are available online at http://hints.cancer.gov.

### 2.2. Measures

#### 2.2.1. Cancer survivorship status

Cancer survivorship was defined using the National Cancer Institute's Office of Cancer Survivorship (OCS) definition and states that “An individual is considered a cancer survivor from the time of cancer diagnosis through the balance of his or her life” [10]. Participants in the HINTS survey were asked the question: “Have you ever been diagnosed as having cancer?” to which response options were “Yes” or “No”. Respondents who answered “Yes” were included in our study and are referred to as “Cancer Survivors” throughout this paper. Respondents who answered “No” were included and stratified analyses were performed by cancer survivorship status.

#### 2.2.2. Study outcome

The main outcome assessed in this study was the source of health information both among cancer survivors and those without a cancer history. To arrive at this outcome, two sequential survey questions were used. First, respondents were asked “Have you ever looked for information about health or medical topics from any source?” to which response options were “Yes” or “No”. Respondents who answered “Yes” were then asked: “The most recent time you looked for information about health or medical topics, where did you go first?” Responses of books, brochures, pamphlets, internet, library, magazines, and newspapers were classified as seekers of health information through media. Our choice of classification was informed by a previous study [11]. Respondents who indicated that they obtained health information from cancer organizations, family, friends/co-workers, doctor/healthcare provider, telephone information number and alternative practitioners were classified as seekers of health information through interpersonal communication. Participants who marked that they have never looked for medical or health information from any source were classified as non-seekers.

#### 2.2.3. Sociodemographic and participant characteristics

Based on previous literature, sociodemographic and participant characteristics assessed in this paper include age (<50, 50–64 years and ≥65 years); gender (Male and Female); race/ethnicity (non-Hispanic white, non-Hispanic black, Hispanics and non-Hispanic other); education (high school degree or less, some college degree or more); time since diagnosis (≤5years, >5 years); employment status (unemployed, employed and retired); income (<$50,000, ≥$50,000); marital status (single, married/living as married, divorced/separated, widowed); having a regular healthcare provider (participants were asked the question “Not including psychiatrists and other mental health professionals, is there a particular doctor, nurse or other health professionals that you see most often?” to which responses were “Yes” or “No”); health insurance coverage (categorized as “Yes” or “No”); general health status (fair/poor, good, excellent/very good); and cancer type (breast, cervical, prostate, colon, rectal, melanoma, other types and more than one type). All cancer type information collected with the questionnaire was included in our study. To improve the power, categories were created for “other cancer types” and “more than one cancer type”. “Other types” category included bladder cancer, bone cancer, endometrial cancer, head and neck cancer, leukemia/blood cancer, liver cancer, lung cancer, lymphoma (Hodgkin's and non-Hodgkin's), oral cancer, ovarian cancer, pancreatic cancer, pharyngeal cancer, renal cancer, stomach cancer and other—specify. Those identified as having non-melanoma skin cancers were excluded from the study.

### 2.3. Statistical Analysis

Analyses were performed using Stata IC 15.1 [12] which accounted for survey sampling weights and the complex sampling design used in HINTS. The study population was made up of 20,365 participants; this included 2412 cancer survivors defined as anyone with a previous history of cancer, who responded to the HINTS survey questionnaire between 2011–2014 and 2017–2018. The other 17,953 were classified as participants without a cancer history. Percentages and confidence intervals were reported based on weighted proportions and thus, results are representative of the population with the characteristics. Covariates with missing data were handled using multiple imputations under the assumption that the data are missing at random [13],[14]. We assessed potential multicollinearity within the covariates using the variance inflation factor (VIF), a measure of the correlation between pairs of variables. The mean VIF in our regression model was 1.87 suggesting that there was no multicollinearity between the variables in the model. A multinomial logistic regression model was used to evaluate the factors associated with sources of health information sought, stratified by cancer survivorship status. Statistical significance was determined using a 2-sided p-value < 0.05 based on Wald test for all comparisons.

## 3. Results

**Figure 1.**
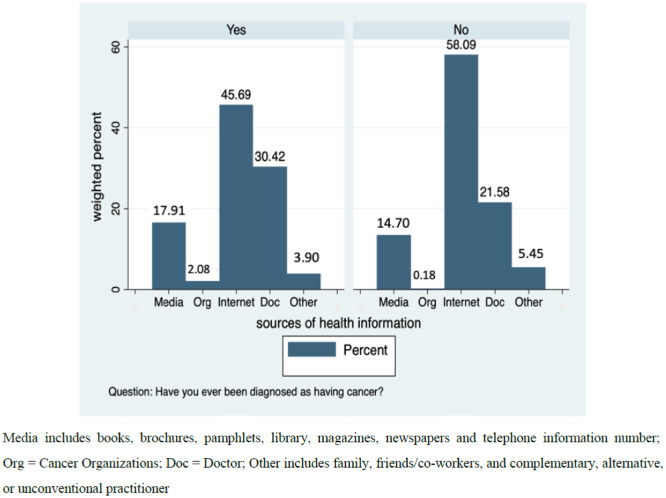
Distribution of Health Information Sources by Cancer Survivorship Status.

Overall, 2412 cancer survivors and 17,953 participants without a cancer history were included in all analyses. The weighted population size of cancer survivors was 15,562,875 while weighted population of those without a cancer history was 218,523,682. Assessment of sociodemographic characteristics of cancer survivors and those without a history of cancer separately, showed that majority of respondents from both groups were female, non-Hispanic white, earning less than $50,000, married with a regular healthcare provider, health insurance coverage and at least some college education. However, while a greater proportion of cancer survivors were 65 years or older, retired, and reported their general health as good, those without a cancer history were mostly younger than 50, employed and reported their general health as excellent or very good (Table 1). Furthermore, though in different proportions, both groups generally first sought health information from the internet. 30.4% of cancer survivors first sought information from their healthcare provider while only 21.6% of respondents without a history of cancer did same (Figure 1).

**Table 1.** Characteristics of Participants by Survivorship Status using HINTS 4 Cycles 1–4 (2011–2014) and HINTS 5 Cycles 1–2 (2017–2018).

	History of Cancer (n = 2412)			No History of Cancer (n = 17,953)		
	wt%	95% CI		wt%	95% CI	
Gender						
Male	38.1	36.1	40.0	39.6	38.9	40.3
Female	61.9	60.0	63.9	60.4	59.7	61.1
Race/Ethnicity						
Non-Hispanic White	71.0	69.0	73.0	59.4	58.7	60.2
Non-Hispanic Black	13.0	11.6	14.4	16.2	15.6	16.7
Hispanic	10.3	9.0	11.6	16.5	15.9	17.0
Non-Hispanic Other	5.7	4.7	6.7	7.9	7.5	8.3
Age						
<50 years	11.4	10.1	12.8	40.0	39.3	40.7
50–64 years	31.5	29.6	33.4	34.3	33.6	35.0
≥65 years	57.1	55.1	59.1	25.7	25.1	26.4
Education						
High School or less	33.8	31.9	35.7	28.8	28.1	29.4
Some College or more	66.2	64.3	68.1	71.2	70.6	71.9
Time since diagnosis						
≤5 years	35.2	33.2	37.2	-	-	-
>5 years	64.8	62.8	66.8	-	-	-
Employment Status						
Unemployed	19.2	17.6	20.9	20.7	20.1	21.3
Employed	29.5	27.6	31.4	54.9	54.2	55.7
Retired	51.3	49.2	53.3	24.4	23.7	25.0
Income						
<$50,000	58.2	56.2	60.2	52.0	51.2	52.7
≥$50,000	41.8	39.8	43.8	48.0	47.3	48.8
Marital Status						
Single	9.3	8.1	10.5	18.4	17.9	19.0
Married/Living as married	50.9	48.8	52.9	52.9	52.2	53.7
Divorced/Separated	21.1	19.5	22.8	19.0	18.4	19.6
Widowed	18.7	17.2	20.3	9.6	9.2	10.1
HealthCare Provider						
Yes	84.4	82.9	85.9	67.7	67.0	68.4
No	15.6	14.1	17.1	32.3	31.6	33.0
General Health						
Fair/Poor	26.8	25.0	28.6	15.9	15.3	16.4
Good	37.6	35.6	39.5	36.0	35.3	36.7
Excellent/Very good	35.6	33.7	37.6	48.1	47.4	48.8
Cancer type						
Breast	20.2	18.6	21.8	-	-	-
Cervical	8.2	7.1	9.3	-	-	-
Prostate	13.3	11.9	14.7	-	-	-
Colon	5.7	4.7	6.6	-	-	-
Rectal	0.5	0.2	0.8	-	-	-
Melanoma	5.9	4.9	6.8	-	-	-
Other	24.3	22.6	26.1	-	-	-
More than one type	21.9	20.3	23.6	-	-	-
Health Insurance						
Yes	94.7	93.8	95.6	89.1	88.6	89.5
No	5.3	4.4	6.2	10.9	10.5	11.4
Information Sources						
Interpersonal Communication	26.1	24.2	28.1	17.0	16.4	17.6
Media	52.7	50.6	54.8	60.9	60.1	61.6
Non-User	21.2	19.5	22.9	22.1	21.4	22.8

Wt = weighted percentage; CI = confidence interval.

Before adjusting for sociodemographic variables and other covariates among cancer survivors, race/ethnicity, age, level of education, income, marital status, having a regular healthcare provider, general health status and cancer type were all associated with sources of health information sought. Compared to non-Hispanic whites (NHWs), Hispanics had lower odds of seeking health information from the media relative to non-seekers (OR: 0.49; 95% CI: 0.29–0.82). Survivors 65 years or older were more likely to seek health information first by interpersonal communication relative to non-seekers (OR: 1.95; 95% CI: 1.02–3.73). Also, a lower level of education was associated with lower odds of health information seeking in cancer survivors either through interpersonal communication or media. Comparing survivors who sought information from the media to non-seekers, media information seekers were more likely to earn $50,000 or more, report very good or excellent health relative to fair/poor health and have higher odds of reporting colon cancer rather than breast cancer. Furthermore, media seekers were less likely to be widowed rather than single when compared to non-seekers. Both health information seekers through the media and interpersonal communication had higher odds of having a regular healthcare provider. (Supplemental Table 1) Among participants without a cancer history, race/ethnicity, age, gender, level of education, employment status, income, marital status, health insurance, having a regular healthcare provider and general health status were associated with seeking health information. Relative to NHWs, non-Hispanic blacks (NHBs), Hispanics and non-Hispanic other (NHO) categories were less likely to seek health information from either media or interpersonal communication compared to non-seekers (Supplemental Table 2).

After adjusting for all possible confounders, the factors associated with sources of health information sought by cancer survivors were age, gender, level of education, income, marital status and having a regular healthcare provider. Compared to cancer survivors aged less than 50 years, those aged ≥65years were 2.8 times more likely to seek this information through interpersonal communication (aOR: 2.80; 95% CI: 1.15–6.82). Cancer survivors with educational levels at or lower than high school education were less likely to seek health information through interpersonal communication (aOR: 0.57; 95% CI: 0.38–0.86) or the media (aOR: 0.35; 95% CI: 0.23–0.51) compared to non-seekers of health information. In addition, males were 39% less likely to seek health information from the media (aOR: 0.61; 95% CI: 0.38–0.99) while those earning $50,000 or more were more likely to (aOR: 1.83; 95% CI: 1.16–2.89). While survivors with a regular health provider had significantly higher odds of seeking health information via interpersonal communication (aOR: 1.92; 95% CI: 1.09–3.38), those who were widowed had lower odds of seeking health information from either interpersonal communication (aOR: 0.28; 95% CI: 0.13–0.60) or the media (aOR: 0.30; 95% CI: 0.13–0.69) (Table 2).

**Table 2.** Weighted, adjusted multinomial logistic regression model predicting factors associated with sources of health information seeking of cancer survivors using HINTS 4 Cycles 1–4 (2011–2014) and HINTS 5 Cycles 1–2 (2017–2018).

	Interpersonal Communication Vs Non-Users			Mass Media Vs Non-Users		
	aOR	CI		aOR	CI	
Race/Ethnicity						
Non-Hispanic White	1.00			1.00		
Non-Hispanic Black	1.16	0.61	2.23	0.73	0.38	1.42
Hispanic	1.01	0.47	2.17	0.81	0.43	1.54
Non-Hispanic Other	2.28	0.89	5.86	0.98	0.43	2.24
Time Since diagnosis						
≤5 years	1.00			1.00		
>5 years	1.21	0.81	1.82	1.43	0.94	2.17
Age						
<50 years	1.00			1.00		
50–64 years	2.25	0.97	5.21	1.53	0.72	3.24
≥65 years	2.80	1.15	6.82	0.97	0.42	2.23
Gender						
Female	1.00			1.00		
Male	0.68	0.41	1.12	0.61	0.38	0.99
Education Level						
Some College or more	1.00			1.00		
High School or less	0.57	0.38	0.86	0.35	0.23	0.51
Employment Status						
Unemployed	1.00			1.00		
Employed	0.59	0.30	1.17	0.71	0.37	1.37
Retired	0.60	0.32	1.13	0.99	0.56	1.73
Income						
< $50,000	1.00			1.00		
$50,000 or more	1.06	0.64	1.75	1.83	1.16	2.89
Marital Status						
Single	1.00			1.00		
Married/Living as married	0.50	0.24	1.02	0.65	0.30	1.37
Divorced	0.45	0.20	1.03	0.66	0.30	1.44
Widowed	0.28	0.13	0.60	0.30	0.13	0.69
Health Insurance						
No	1.00			1.00		
Yes	1.19	0.33	4.22	0.93	0.26	3.31
Regular provider						
No	1.00			1.00		
Yes	1.92	1.09	3.38	1.74	0.99	3.06
General health						
Fair/Poor	1.00			1.00		
Good	0.94	0.59	1.48	0.83	0.54	1.28
Excellent/Very good	1.12	0.68	1.83	1.28	0.82	2.00
Cancer type						
Breast Cancer	1.00			1.00		
Cervical Cancer	0.63	0.25	1.60	0.63	0.27	1.51
Prostate Cancer	1.15	0.52	2.56	0.80	0.37	1.76
Colon Cancer	0.78	0.29	2.11	0.51	0.22	1.22
Rectal Cancer	3.26	0.25	41.86	6.79	0.68	67.83
Melanoma	0.79	0.29	2.14	0.80	0.33	1.93
Other types	0.72	0.38	1.40	0.72	0.38	1.35
More than one type	1.03	0.50	2.15	0.67	0.33	1.38

aOR = Adjusted Odds Ratio; CI = confidence interval; Model adjusted for race/ethnicity, time since cancer diagnosis, age, gender, educational level, employment status, income, marital status, health insurance, having a regular healthcare provider, general health status and cancer type.

Among participants without a history of cancer, the factors associated with sources of health information sought first were race/ethnicity, age, gender, level of education, income, marital status and having a regular healthcare provider. Compared to NHWs, NHBs (aOR: 0.54; 95% CI: 0.44–0.66), Hispanics (aOR: 0.49; 95% CI: 0.41–0.60) and NHO (aOR: 0.54; 95% CI: 0.40–0.72) categories were significantly less likely to seek health information from the media. While males and those with education levels at or lower than high school had lower odds of seeking health information using either interpersonal communication or the media, those with a regular health provider had higher odds of seeking health information from both sources relative to non-seekers. Participants 65 or older were less likely to seek information from the media (aOR: 0.52; 95% CI: 0.41–0.67) while those 50 to 64 years were more likely to seek information through interpersonal communication (aOR: 1.25; 95% CI: 1.00–1.56). Those who were married had higher odds of seeking information from the media (aOR: 1.27; 95% CI: 1.05–1.54) while those who were widowed had lower odds of doing same (aOR: 0.60; 95% CI: 0.45–0.78). In addition, earning an income of $50,000 or higher was associated with seeking information from media sources (aOR: 1.72; 95% CI: 1.41–2.10) (Table 3).

**Table 3.** Weighted, adjusted multinomial logistic regression model predicting factors associated with sources of health information seeking of participants without a history of cancer using HINTS 4 cycles 1–4 and HINTS 5 cycles 1–2 (2011–2018).

	Interpersonal Communication Vs Non-Users			Mass Media Vs Non-Users		
	OR	CI		OR	CI	
Race/Ethnicity						
Non-Hispanic White	1.00			1.00		
Non-Hispanic Black	0.90	0.70	1.16	0.54	0.44	0.66
Hispanic	0.85	0.67	1.09	0.49	0.41	0.60
Non-Hispanic Other	0.87	0.62	1.21	0.54	0.40	0.72
Age						
<50 years	1.00			1.00		
50–64 years	1.25	1.00	1.56	0.85	0.72	1.01
≥65 years	1.28	0.98	1.67	0.52	0.41	0.67
Gender						
Female						
Male	0.66	0.55	0.80	0.53	0.46	0.61
Education Level						
Some College or more	1.00			1.00		
High School or less	0.62	0.51	0.76	0.29	0.25	0.35
Employment Status						
Unemployed	1.00			1.00		
Employed	0.80	0.63	1.03	1.02	0.82	1.28
Retired	0.91	0.70	1.19	1.05	0.81	1.36
Income						
<$50,000	1.00			1.00		
$50,000 or more	1.16	0.92	1.47	1.72	1.41	2.10
Marital Status						
Single	1.00			1.00		
Married/Living as married	1.28	0.99	1.65	1.27	1.05	1.54
Divorced	0.87	0.66	1.15	0.95	0.76	1.18
Widowed	0.91	0.67	1.23	0.60	0.45	0.78
Health Insurance						
No	1.00			1.00		
Yes	1.20	0.88	1.63	0.96	0.77	1.21
Regular provider						
No	1.00			1.00		
Yes	1.84	1.50	2.26	1.46	1.24	1.72
General health						
Fair/Poor	1.00			1.00		
Good	0.93	0.73	1.19	1.12	0.90	1.38
Excellent/Very good	0.81	0.62	1.06	0.99	0.79	1.23

aOR = Adjusted Odds Ratio; CI = confidence interval; Model adjusted for race/ethnicity, age, gender, educational level, employment status, income, marital status, health insurance, having a regular healthcare provider and general health status

## 4. Discussion

Findings of the index study indicate that health information seeking behaviors are prevalent among cancer survivors, with about 80% of cancer survivors reporting that they had sought health information from several sources. Remarkably, a majority of cancer survivors report seeking health information from the internet before considering other sources, with only 30% of the population first consulting their healthcare provider for health information. Our findings are in contrast to an earlier study where a majority of cancer survivors reported first consulting their health providers for health information [4]. The results were similar among participants without a history of cancer as about the same proportion seek health information from several sources with majority reporting the use of media as their first source of health information. This is to be expected because of the availability of various media sources, especially the internet, and its easy accessibility for searches before an appointment with a health care provider can be scheduled. However, a smaller proportion of cancer survivors were found to seek health information from the internet compared to non-cancer participants and this may be due to the frequent consultations and hospital encounters that cancer survivors typically have. Our study provides more up-to-date information and is suggestive of changing patterns of health information seeking behaviors among cancer survivors. These findings are largely corroborated by recent literature that point to the internet as a predominant and primary source of health information in this population [6],[15],[16].

In both cancer survivors and participants without a cancer history, regardless of the source of information, respondents who had an education at the level of high school or less were significantly less likely to seek health information. This is in line with other studies reporting this finding among respondents in the general US population. [17]–[20] Our study shows that this also holds true for cancer survivors, further emphasizing the role of education in health information seeking behaviors. In addition, we found that male cancer survivors are less likely to seek health information from the media while males without a cancer history are less likely to seek health information through media or interpersonal communication. The literature has already established that men are less likely to seek health care than women and this negatively impacts their health. [6],[21],[22] The finding that they also do not readily seek health information potentially worsens the problem and exposes the need for educational interventions focused on this population regardless of survivorship status. Not seeking healthcare or health information may leave them oblivious of the state of their health and increase their risk of developing ailments they are largely unaware of until the disease has significantly progressed.

Among cancer survivors, respondents who identified as widowed were less likely to seek health information whether through interpersonal communication or the media. Also, survivors 65 years or older were more likely to seek health information through interpersonal communication. This suggests that these key sociodemographic factors are pervasive in their relationship with health information seeking behaviors among cancer survivors. Our study findings are supported by other studies that have found age and marital status to be associated with health information seeking behaviors. [6],[23] Adjei Boakye et al found that among participants without a cancer diagnosis, identifying as widowed was associated with a lower likelihood of seeking out health information. Our study has been able to identify that within this population, widows are less likely to seek health information from the media (including the internet) but do not have any lower likelihood of seeking information through interpersonal communication [6]. On the other hand, widowed cancer survivors were less likely to seek health information from any source, in contrast to findings in the literature [6]. Previous studies have reported that widows have a higher risk of mortality, are more likely to have poorer health and have higher healthcare utilization than those who are married [24]–[26]. This, coupled with a past cancer diagnosis, suggests that they have more health issues than their average married counterparts and need to be more active in seeking out health information to improve their health. This population ought to be specifically targeted to improve their health information seeking behaviors.

Individuals who earned $50,000 and above were more likely to obtain health information from media sources regardless of cancer survivorship status. Given the expected absence of socioeconomic barriers in this population, it is plausible that this group has a decent amount of exposure to media tools. This may have boosted their self-perceived efficacy in obtaining health information from the pertinent media sources. On the other hand, though race/ethnicity was identified as a factor affecting health information seeking through the media in respondents without a cancer diagnosis, these racial differences were not seen in cancer survivors. This may be explained by the dynamics of cancer treatment and engagement with the healthcare system leading to more enlightenment of cancer survivors about the need for health information regardless of their race/ethnicity. Survivors who reported having a regular healthcare provider were more likely to first seek health information through interpersonal communication. Conversely, those without a cancer diagnosis who had a regular healthcare provider were more likely to seek health information from both sources. This finding may be explained by the results of a study which showed that most cancer survivors first consulted the internet for health information even though a majority preferred to obtain health information from their health care provider first [7]. Cancer diagnosis is undoubtedly stressful and may help build trust between the survivor and their healthcare provider so that though they consult the internet, they still seek health information through interpersonal communication to validate their experiences.

Cancer survivors are predisposed to developing several comorbidities/comorbid conditions [27], as well as additional cancers [28]–[30], hence it is crucial that they obtain quality and accurate health information. Most cancer patients have co-morbid conditions with long term sequelae and associated care, cutting across multiple disciplines. This is usually accompanied by an array of continuous laboratory monitoring, work-up, diagnostics as well as complex procedures and medications for their management. Having the right source of health information may increase the chances that patients would comply with their management plan and get optimal care. Additionally, our study reveals that about a fifth of cancer survivors do not seek any source of health information. This is worrisome as it may have potential impact in the management and clinical outcomes of this group of cancer survivors. While health information sources are readily available through various channels, this finding further emphasizes the need for focused interventions to ensure that non-seekers are reached with the right information they need to improve their health outcomes. Findings of the present study identify sources of health information that can potentially be leveraged toward ensuring cancer survivors are exposed to accurate health information. They also point to some other sources whose potentials are not being utilized maximally. For example, cancer organizations have been shown to be very useful in the dissemination of cancer-related health information, as well as support for cancer survivors [31],[32] however our study revealed that less than 5% of cancer survivors rely on this as a source of health information. This calls for increased efforts toward activities that empower cancer support organizations.

Given that cancer survivors have frequent routine clinical encounters with their healthcare providers, every clinic visit should be maximized to ensure that the right information is provided, and a system for the continuous provision of health information is initiated. For example, the use of interactive mobile health program and automated messaging emanating from health providers could help disseminate and reinforce the right information that is relevant to patients at every stage of their management or direct them to verified sites where the right information could be sourced. Previous studies have shown that most patients are receptive to electronic communication with their healthcare providers [33],[34]. However, further studies could evaluate the impact of this strategy in bridging the gap in cancer survivors, particularly among those that are non-seekers of health information.

## 5. Strengths and limitations

Our study has several strengths. First, our findings are derived from pooling several years of the HINTS data, which boosts overall sample size and ensures for a more robust analysis and precise findings. More so, pooling data across time provides an indication of what practices have been more predominant within the period in question. In addition, HINTS data is representative of the non-institutionalized US population and so our findings are generalizable. Nonetheless, this study is not without limitations. Chief among these is the issue of low response rates to the HINTS. However, rigorous sampling techniques and weighting procedures were applied by HINTS investigators to account for this low response rate, hence limiting the potential for resultant bias. Regardless, our study still has the potential for recall bias. HINTS data is based on a cross-sectional study design, and as such, causal inferences cannot be made from our analysis. Therefore, longitudinal studies that demonstrate a temporal sequence and account for changes in health seeking behavior over time is crucial. Furthermore, cancer organizations as a source of health information could have been further explained. From the dataset used, it was difficult to tell whether this included cancer organizational websites or if those who used these websites identified as internet seekers, thus causing them to be categorized as media seekers. Cancer organizations provide information through multiple sources, but this was not captured in the data with potential overlap in health information channels. In any case, it is still important to highlight that cancer organizations put out valuable information which should be utilized to the uttermost by the cancer survivor population.

In conclusion, our study generated findings that predict health information seeking behaviors through the media and interpersonal communication both in cancer survivors and in individuals without a cancer diagnosis. We also found that less than 5% of cancer survivors seek health information from cancer organizations. Activities that improve the visibility of cancer organizations among cancer-surviving populations should be enhanced to help cancer survivors take advantage of the vast amount of health information available from these sources. In addition, 21% of cancer survivors do not seek health information from any source while socioeconomic status, age, and gender were identified as important correlates of health information source choice among cancer survivors in the US. We also compared the findings among cancer survivors to those without a cancer diagnosis. These factors may be useful in guiding health communication interventions aimed at various groups of cancer surviving populations to ensure that they improve their health seeking behaviors.
