# Supplementary material for: Sources of health information among U.S. cancer survivors: results from the health information national trends survey (HINTS)
Source: AIMS Public Health. 2020 Jun 12;7(2):363–79. doi: 10.3934/publichealth.2020031 (PMC7327406; doi:10.3934/publichealth.2020031)
Supplement: Supplementary file 1 [file publichealth-07-02-031-s001.pdf]

Public Health

AIMS Public Health, 7(2): 363–379.
DOI: 10.3934/publichealth.2020031
Received: 20 April 2020
Accepted: 10 June 2020
Published: 12 June 2020

http://www.aimspress.com/journal/aimsph

Research article

Sources of health information among U.S. cancer survivors: results from the health information national trends survey (HINTS)

Inimfon Jackson[1,*], Ikponmwosa Osaghae[1], Nnenna Ananaba[2], Aniekeme Etuk[3], Nsikak Jackson[3] and Onyema G Chido-Amajuoyi[4]

[1] Department of Epidemiology, Human Genetics and Environmental Sciences, University of Texas School of Public Health, University of Texas Health Science Center at Houston, Houston, TX, USA
[2] Department of Health Promotion and Behavioral Sciences, University of Texas School of Public Health, University of Texas Health Science Center at Houston, Houston, TX, USA
[3] Department of Management, Policy and Community Health, University of Texas School of Public Health, University of Texas Health Science Center at Houston, Houston, TX, USA
[4] Department of Epidemiology, University of Texas MD Anderson Cancer Center, Houston, TX, USA

* **Correspondence:** Email: Inimfon.N.Jackson@uth.tmc.edu; Tel: +16672121139.

Appendix

Supplemental Table 1. Unadjusted multinomial logistic regression model predicting factors associated with sources of health information seeking of cancer survivors using HINTS 4 Cycles 1–4 (2011–2014) and HINTS 5 Cycles 1–2 (2017–2018).

	Interpersonal Communication Vs Non-Seekers				Media Vs Non-Seekers			
	OR	CI		p value	OR	CI		p value
Race/Ethnicity								
Non-Hispanic White	1.00				1.00			
Non-Hispanic Black	1.09	0.63	1.89	0.746	0.63	0.36	1.11	0.109
Hispanic	0.72	0.39	1.35	0.311	0.49	0.29	0.82	0.007
Non-Hispanic Other	1.54	0.70	3.43	0.283	0.66	0.33	1.29	0.222
Time Since diagnosis								
≤5 years	1.00				1.00			
> 5 years	1.14	0.77	1.69	0.521	1.14	0.80	1.64	0.468
Age								
< 50 years	1.00				1.00			
50 - 64 years	1.97	0.98	3.96	0.056	1.48	0.81	2.70	0.201
≥ 65 years	1.95	1.02	3.73	0.043	0.77	0.44	1.34	0.358
Gender								
Female	1.00				1.00			
Male	0.92	0.64	1.33	0.655	0.77	0.56	1.07	0.125
Education Level								
Some College or more	1.00				1.00			
High School or less	0.57	0.39	0.83	0.003	0.25	0.17	0.36	0.000
Employment Status								
Unemployed	1.00				1.00			
Employed	0.78	0.42	1.44	0.428	1.43	0.85	2.43	0.180
Retired	0.90	0.51	1.59	0.715	0.95	0.61	1.49	0.830
Income								
< $50,000	1.00				1.00			
$50,000 or more	1.29	0.83	2.02	0.260	3.00	2.03	4.44	0.000
Marital Status								
Single	1.00				1.00			
Married/Living as married	0.68	0.36	1.28	0.229	0.91	0.49	1.71	0.775
Divorced	0.58	0.28	1.20	0.141	0.65	0.34	1.26	0.207
Widowed	0.45	0.23	0.87	0.017	0.32	0.16	0.62	0.001
Health Insurance								
No	1.00				1.00			
Yes	1.67	0.67	4.16	0.271	1.54	0.72	3.32	0.266

Continued on next page

	Interpersonal Communication Vs Non-Seekers				Media Vs Non-Seekers			
	OR	CI		p value	OR	CI		p value
Regular provider								
No	1.00				1.00			
Yes	1.68	1.02	2.75	0.041	2.09	1.40	3.13	0.000
General health								
Fair/Poor	1.00				1.00			
Good	0.88	0.58	1.35	0.569	1.18	0.79	1.74	0.422
Excellent/Very good	1.18	0.77	1.80	0.456	2.22	1.50	3.29	0.000
Cancer type								
Breast Cancer	1.00				1.00			
Cervical Cancer	0.64	0.27	1.49	0.298	0.83	0.41	1.68	0.605
Prostate Cancer	1.03	0.56	1.92	0.917	0.62	0.36	1.07	0.086
Colon Cancer	0.86	0.34	2.17	0.744	0.46	0.22	0.97	0.042
Rectal Cancer	4.12	0.35	48.41	0.259	7.16	0.73	70.63	0.092
Melanoma	0.67	0.28	1.60	0.372	1.03	0.50	2.12	0.931
Other types	0.69	0.39	1.22	0.197	0.72	0.43	1.21	0.218
More than one type	1.14	0.61	2.12	0.686	0.76	0.43	1.34	0.343

OR = Odds Ratio; CI = confidence interval

Supplemental Table 2. Unadjusted multinomial logistic regression model predicting factors associated with sources of health information seeking of participants without a cancer history using HINTS 4 Cycles 1–4 (2011–2014) and HINTS 5 Cycles 1–2 (2017–2018).

	Interpersonal Communication Vs Non-Seekers				Media Vs Non-Seekers			
	OR	CI		p value	OR	CI		p value
Race/Ethnicity								
Non-Hispanic White	1.00				1.00			
Non-Hispanic Black	0.77	0.60	0.98	0.034	0.45	0.37	0.54	0.000
Hispanic	0.66	0.52	0.83	0.000	0.38	0.33	0.45	0.000
Non-Hispanic Other	0.78	0.57	1.06	0.110	0.59	0.46	0.76	0.000
Age								
<50 years	1.00				1.00			
50–64 years	1.40	1.14	1.73	0.002	0.94	0.81	1.09	0.445
≥65 years	1.62	1.35	1.95	0.000	0.51	0.45	0.59	0.000
Gender								
Female	1.00				1.00			
Male	0.65	0.55	0.78	0.000	0.62	0.55	0.71	0.000
Education Level								
Some College or more	1.00				1.00			
High School or less	0.62	0.52	0.73	0.000	0.22	0.19	0.26	0.000
Employment Status								
Unemployed	1.00				1.00			
Employed	0.89	0.72	1.10	0.263	1.52	1.27	1.82	0.000
Retired	1.32	1.07	1.64	0.010	0.87	0.72	1.04	0.122
Income								
< $50,000	1.00				1.00			
$50,000 or more	1.36	1.13	1.64	0.001	2.82	2.43	3.26	0.000
Marital Status								
Single	1.00				1.00			
Married/Living as married	1.56	1.23	1.96	0.000	1.48	1.26	1.75	0.000
Divorced	1.04	0.79	1.37	0.768	0.85	0.69	1.05	0.135
Widowed	1.47	1.12	1.93	0.006	0.45	0.36	0.56	0.000
Health Insurance								
No	1.00				1.00			
Yes	1.79	1.36	2.35	0.000	1.62	1.35	1.96	0.000
Regular provider								
No	1.00				1.00			
Yes	2.26	1.87	2.74	0.000	1.77	1.54	2.03	0.000

Continued on next page

	Interpersonal Communication Vs Non-Seekers				Media Vs Non-Seekers			
	OR	CI		p value	OR	CI		p value
General health								
Fair/Poor	1.00				1.00			
Good	0.97	0.77	1.22	0.802	1.57	1.30	1.89	0.000
Excellent/Very good	0.90	0.72	1.14	0.384	1.89	1.57	2.27	0.000

OR = Odds Ratio; CI = confidence interval.

AIMS Press

© 2020 the Author(s), licensee AIMS Press. This is an open access article distributed under the terms of the Creative Commons Attribution License (http://creativecommons.org/licenses/by/4.0)
